# Monitoring the health of transitioning professional footballers: protocol of an observational prospective cohort study

**DOI:** 10.1136/bmjsem-2019-000680

**Published:** 2019-12-15

**Authors:** Vincent Gouttebarge, Thor Einar Andersen, Charlotte Cowie, Edwin Goedhart, Harald Jorstad, Simon Kemp, Marsh Königs, Mario Maas, Emmanuel Orhant, Jussi Rantanen, Jari Salo, Luis Serratosa, Keith Stokes, Johannes L Tol, Evert Verhagen, Alexis Weber, Gino Kerkhoffs

**Affiliations:** 1 Amsterdam UMC, Univ of Amsterdam, Department of Orthopaedic Surgery, Amsterdam Movement Sciences, Meibergdreef 9, Amsterdam, The Netherlands; 2 FIFPRO (Football Players Worldwide), Hoofddorp, The Netherlands; 3 Academic Center for Evidence based Sports medicine (ACES), Amsterdam Movement Sciences, Amsterdam, The Netherlands; 4 Amsterdam Collaboration on Health & Safety in Sports (ACHSS), Amsterdam UMC IOC Research Center of Excellence, Amsterdam, The Netherlands; 5 Division of Exercise Science and Sports Medicine, University of Cape Town, Cape Town, South Africa; 6 Oslo Sports Trauma Research Center, Department of Sports Medicine, Norwegian School of Sport Sciences, Oslo, Norway; 7 The Norwegian FA Medical Center, The Football Association of Norway, Oslo, Norway; 8 The Football Association, National Football Centre, St George’s Park, Needwood, United Kingdom; 9 Royal Netherlands Football Association (KNVB), FIFA Medical Center of Excellence, Zeist, The Netherlands; 10 Amsterdam UMC, Univ of Amsterdam, Department of Cardiology, Amsterdam Movement Sciences, Meibergdreef 9, Amsterdam, The Netherlands; 11 Rugby Football Union, Twickenham, UK; 12 Emma Children’s Hospital, Amsterdam UMC, University of Amsterdam, Emma Neuroscience Group, Amsterdam, The Netherlands; 13 Amsterdam UMC, Univ of Amsterdam, Department of Musculoskeletal Radiology, Amsterdam Movement Sciences, Meibergdreef 9, Amsterdam, The Netherlands; 14 French Football Federation (FFF), Clairefontaine Medical Centre, FIFA Medical Center of Excellence, Clairefontaine, France; 15 Orthopaedics and Sports Clinic, Mehiläinen NEO Hospital, Turku, Finland; 16 Sports Hospital Mehiläinen, Helsinki, Finland; 17 Ripoll & De Prado Sport Clinic, FIFA Medical Centre of Excellence, Madrid, Spain; 18 Hospital Universitario Quironsalud, Madrid, Spain; 19 Department for Health, University of Bath, Bath, United Kingdom; 20 Centre for Sport, Exercise and Osteoarthritis Research Versus Arthritis, University of Bath, Bath, United Kingdom; 21 Amsterdam UMC, Vrije Universiteit Amsterdam, Department of Public and Occupational Health, Amsterdam Movement Sciences, de Boelelaan 1117, Amsterdam, The Netherlands; 22 Fédération Internationale de Football Association (FIFA), Zurich, The Netherlands

**Keywords:** football, osteoarthritis, cardiovascular epidemiology, neurology, psychology

## Abstract

**Introduction:**

Transitioning out of professional football is a challenging time in most players’ lives. During these preretirement and postretirement years, professional footballers may struggle with their mental, musculoskeletal, neurocognitive and cardiovascular health. Currently, longitudinal data about these health conditions are lacking. This article presents the design of a prospective cohort study with the primary aim of gathering epidemiological evidence about the onset and course of mental, musculoskeletal, neurocognitive and cardiovascular health conditions in professional footballers during their preretirement and postretirement years and evaluating the associations between risk indicators and the health conditions under study in these players.

**Methods and analysis:**

An observational prospective cohort study with repeated measurements over a follow-up period of 10 years will be conducted among at least 200 professional footballers (male; 27 (±1) years old). Mental health will be explored by assessing symptoms of distress, anxiety, depression, sleep disturbance, alcohol misuse, drug misuse and disordered eating. Musculoskeletal health will be explored by assessing severe joint injury and related surgery, clinical and radiological osteoarthritis, and joint function (hips, knees and ankles). Neurocognitive health will be explored by assessing the concussion, brain structure and functioning, and neurocognitive functioning. Cardiovascular health will be explored by assessing blood pressure, lipid profile and ECG abnormalities.

**Ethics and dissemination:**

Ethical approval for the study was provided by the Medical Ethics Review Committee of the Amsterdam University Medical Centers. The results of the study will be submitted to peer-reviewed journals, will be presented at scientific conferences and will be released in the media (postpublication).

**Trial registration number:**

The Dutch Trial Registry (Drake Football Study NL7999).

## Introduction

Transitioning out of professional football is known to be a significant and challenging time in most male players’ lives.^1 2^ During these preretirement and postretirement years, professional footballers may face several challenges such as preparing for their future occupational career and adjusting to a new identity, life and lifestyle. Also, they are likely to experience problems with their mental, musculoskeletal, neurocognitive and cardiovascular health.^2 3^ Mental health symptoms are common in both active and retired professional footballers, being related to various stressors such as severe injuries and related surgeries, concussions, transitioning between clubs and ultimately out of sport and postsport chronic pain.^4–9^ Osteoarthritis (OA) in the ankle, knee and hip joints occurs more frequently in retired professional footballers than in the general population (matched for gender and age), being especially related to severe traumatic injuries and related surgeries during their career.^10–14^ Furthermore, a recent study involving 964 active and 396 retired professional footballers showed that the prevalence of knee OA linearly increases through the preretirement and postretirement years.^15^ When it comes to neurocognition, several studies have suggested that recently retired professional footballers might have impairments in neurocognitive function (eg, attention, memory and concentration) when compared with age-matched controls, although the clinical significance of these findings remains unclear.^16–18^ Exposure to vigorous exercise training during an elite sport career is associated with lower cardiovascular morbidity and mortality as well as with higher life expectancy.^19 20^ By contrast, several cross-sectional studies have reported an increased prevalence of some cardiovascular risk factors in active and retired professional athletes, for instance, hypertension, hyperlipidaemia and impaired fasting glucose, while others suggest that lifelong intense exercise might be associated with cardiac irregularities such as a higher prevalence of atrial fibrillation and more coronary atherosclerotic plaques.^21–24^ Whether irregular cardiac activities occur and how they evolve in professional footballers during their preretirement and postretirement years remains unclear.

Established principally from cross-sectional studies, this epidemiological evidence warrants prospective cohort studies in order to further clarify the onset and course of any mental, musculoskeletal, neurocognitive and cardiovascular health conditions among professional footballers during their preretirement and postretirement years. Accordingly, we designed a prospective cohort study with a follow-up period covering prefootball and postfootball years, with the following descriptive and analytic objectives: (descriptive objective) to gather epidemiological evidence about the onset and course of mental, musculoskeletal, neurocognitive and cardiovascular health conditions (dependent variables) in professional footballers during their preretirement and postretirement years, comparing them to matched controls from the non-elite sport population (if applicable) and (analytical objective) to evaluate the associations between risk indicators (independent variables) and the onset and course of mental, musculoskeletal, neurocognitive and cardiovascular health conditions (dependent variables) in professional footballers during their preretirement and postretirement years. The hypotheses are that (descriptive objective) the health of professional footballers differs from that of matched controls (non-elite sport population), and that (analytical objective) professional footballers who are exposed to a risk indicator (or a combination of risk indicators) have a higher risk of developing a mental, musculoskeletal, neurocognitive and cardiovascular health condition than those unexposed. This article describes the design of our prospective cohort study.

## Methods and analysis

### Study design

An observational prospective cohort study with repeated measurements over a follow-up period of 10 years will be conducted in accordance with the Declaration of Helsinki and guided by the ‘Strengthening the Reporting of Observational Studies in Epidemiology’ statement.^25 26^

### Study participants

Participants must fulfil the following inclusion criteria: (1) male; (2) professional footballer; (3) member of Football Players Worldwide (FIFPRO) and affiliated national unions; (4) 27 (±1) years old at the start of the study and (5) able to read and comprehend texts in English (in addition to participants’ native language). In our study, the definition of a professional footballer is that he: (1) trains to improve football performances; (2) competes in the highest or second highest national league in his country and (3) has football training and competition as his main occupational activity. In our study, we will include participants between 26 and 28 years old in order to monitor their health during their preretirement and postretirement years, under the assumption that professional footballers retire on average at around 32 years old.^6^

### Dependent variables: health conditions and instruments

#### Mental health conditions

Mental health will be explored by assessing symptoms of distress, anxiety, depression, sleep disturbance, alcohol misuse, drug misuse and disordered eating.^27^ These assessments related to mental health take around 20 min to be completed.

- Distress: In the preretirement years, distress in the previous 4 weeks is assessed using the Athlete Psychological Strain Questionnaire (APSQ) based on 10 items (eg, ‘During the past 4 weeks, I could not stop worrying about injury or my performance’) scored on a 5-point scale (from ‘none of the time’ to ‘all of the time’).^28^ The APSQ has been recently validated in the athletic population (internal consistency: 0.5–0.9; criterion-related validity: area under ROC curve >0.9).^28 29^ A total score ranging from 10 to 50 is obtained by summing up the answers on the 10 items, a score of 17 or more indicating an elevated or high risk for (athletic) distress.^28 29^ In the postretirement years, distress in the previous 4 weeks is assessed using the widely used and validated Kessler-10 based on 10 items (eg, ‘During the past 4 weeks, about how often did you feel hopeless?’) scored on a 5-point scale (from ‘none of the time’ to ‘all of the time’).^30^ A total score ranging from 10 to 50 is obtained by summing up the answers on the 10 items, a score of 25 or more indicating a moderate or severe level of (psychological) distress.^30^
- Anxiety: The 7-item General Anxiety Disorder-7 (GAD-7) is used to assess symptoms related to anxiety in the previous 4 weeks (eg, ‘Have you been feeling nervous, anxious, or on edge?’) scored on a 4-point scale (from ‘not at all’ to ‘nearly every day’).^31^ The GAD-7 has been validated in several populations and European languages (internal consistency: 0.9; test–retest reliability: 0.8; criterion-related validity: sensitivity 0.9, specificity 0.8, area under ROC curve >0.9).^31^ A total score ranging from 0 to 21 is calculated by summing up the answers on the seven items, with a score of 10 or more indicating the presence of moderate anxiety.^31^
- Depression: The Patient Health Questionnaire-9 (PHQ-9) is used to assess the presence of symptoms of depression in the previous 4 weeks (eg, ‘Have you been feeling down, depressed or hopeless?’) scored on a 4-point scale (from ‘not at all’ to ‘nearly every day’).^32 33^ The PHQ-9 has been validated in several populations and European languages (internal consistency: >0.8; criterion-related validity: sensitivity >0.8, specificity >0.8, area under ROC curve >0.9).^32 33^ A total score ranging from 0 to 27 is calculated by summing up the answers on the nine items, with a score of 10 or more indicating the presence of moderate depression.^32 33^
- Sleep disturbance: Measured using the shortened Athlete Sleep Screening Questionnaire (ASSQ), sleep disturbance in the previous 4 weeks is assessed through five items (eg, ‘How satisfied/dissatisfied are you with the quality of your sleep?’) scored on 4-point and 5-point scales.^34 35^ The ASSQ has been validated in athletes (internal consistency: >0.7; test–retest reliability: >0.8; criterion-related validity: sensitivity >0.8, specificity >0.9).^34 35^ A total score ranging from 1 to 17 is obtained by summing up the answers to the five items, a score of 8 or more indicating the presence of moderate sleep disturbance.^34 35^
- Alcohol misuse: Level of alcohol consumption is detected using the validated 3-item Alcohol Use Disorders Identification Test (AUDIT-C; eg, ‘How many standard drinks containing alcohol do you have on a typical day?’).^36^ The AUDIT-C has been validated in several populations and European languages (test–retest coefficients: 0.6–0.9; criterion-related validity: area under ROC curve 0.70-<1.0).^36 37^ A total score ranging from 0 to 12 is obtained by summing up the answers on the three items, a score of 5 or more indicating the presence of alcohol misuse.^36^
- Drug misuse: Based on the Cutting down, Annoyance by criticism, Guilty feeling and Eye-openers Adapted to Include Drugs (CAGE-AID), drug(s) misuse in the previous 3 months is assessed through four items (eg, ‘In the last 3 months, has anyone annoyed you or gotten on your nerves by telling you to cut down or stop using drugs?’) scored as yes or no.^38 39^ The CAGE-AID has been validated in several populations and European languages (reliability: >0.9; sensitivity: >79%; specificity: >97%).^38 39^ A total score ranging from 0 to 4 is obtained by summing up the answers to the four questions, a score of 2 or more indicating the presence of drug misuse.^38 39^
- Disordered eating: The Brief Eating Disorder in Athletes Questionnaire (BEDA-Q) is used to assess the presence of disordered eating in the previous 4 weeks through nine items (eg, ‘I feel extremely guilty after overeating’) scored on several scales.^40^ The BEDA-Q has been validated in athletes (internal consistency: >0.8; criterion-related validity: sensitivity >0.8, specificity >0.8, area under ROC curve >0.7).^40^ A total score ranging from 0 to 18 is calculated by summing up the answers on the first six items, with a score of 2 or more indicating the presence of disordered eating.^41^

#### Musculoskeletal health conditions

Musculoskeletal health will be explored by assessing the following outcomes in the ankles, knees and hips: severe joint injury and related surgery, clinical and radiological OA, knee cartilage quality and joint function. These assessments related to musculoskeletal health take around 40 min to be completed.

- Severe joint injury and related surgery: The number of severe injuries and surgeries in the ankles, knees and hips is assessed through single questions. Football-related injuries and surgeries are assessed in the preretirement years, non-football-related (eg, traffic accident) injuries and surgeries in the preretirement and postretirement years and sport-related injuries and surgeries in the postretirement years. In our study, football-related and sport-related severe joint injury is defined as an injury that involves the joint (ankle, knee and hip), occurs during football or sport activities and leads to either training or match absence for more than 28 days (definition being clearly stated to the participants).^42^ For these questions, participants are recommended to consult either their medical record or their physician.
- Clinical OA: The presence of clinical OA in the ankles, knees and hips is assessed by a physician, based on both history and physical examination. According to the adapted criteria of the National Institute for Health and Care Excellence, OA is diagnosed in cases of (1) activity-related joint pain; (2) restricted range of motion (ROM) of the joint and (3) either no morning joint-related stiffness or morning stiffness that lasts no longer than 30 min.^43^
- Radiological OA: Two-sided weight-bearing radiographs are performed (ankles: mortise, anteroposterior and lateral views; knees: Rosenberg, standing anteroposterior and lateral views; hips: anteroposterior pelvic views).^44–46^ OA is diagnosed and severity classified (none, mild, moderate/severe) according to Kellgren and Lawrence criteria (normal, grade 1, grade 2, grade 3 and grade 4).^47^
- Knee cartilage quality: To obtain cartilage and menisci morphology and lesions, cone beam CT (CBCT) of the knees is performed, with a knee actively flexed and extended with free ROM and a belt containing standard hydroxyapatite phantoms placed around the knees at the height of proximal tibia.^48 49^ Because CBCT as an imaging technique is not likely to be available everywhere, only a subgroup of participants will be invited to this assessment.
- Joint function: The validated American Academy of Orthopedic Surgeons (AAOS) foot and ankle questionnaire is used to assess the level of foot and ankle-related function.^50^ Based on the score on 25 items, each measured on a 5 or 6-point scale and subsequently entered into a computerised AAOS spreadsheet, a total score ranging from 0 to 100 is calculated where higher scores indicate better foot and ankle function.^50^ The Knee injury and Osteoarthritis Outcome Score Physical Function Short Form (KOOS-PS) is used to assess the level of physical knee function.^51 52^ The KOOS-PS has been validated in several study populations and European languages.^51–53^ Based on the score on seven items, each measured on a 5-point scale (from 0 to 4) and subsequently converted, a total score ranging from 0 to 100 is calculated, where 0 represents total knee disability and 100 perfect knee function.^52^ The Hip dysfunction and Osteoarthritis Outcome Score Shortform is used to assess hip-related pain and impaired activities.^54 55^ Based on the score on five items measured on a 5-point scale (from 0 to 4) and subsequently converted, a total score ranges from 0 to 100, where 0 represents total hip disability and 100 represents perfect hip health.^55^

#### Neurocognitive health conditions

Neurocognitive health will be explored by assessing concussion (including detailed history and neurological consequences), brain structure and functioning, and neurocognitive functioning. These assessments related to neurocognitive health take around 45 min to be completed.

- Concussion: The number of concussions is examined through a single question. Football-related concussions are assessed in the preretirement years, non-sports-related (eg, traffic accident) concussions in the preretirement and postretirement years and sport-related concussions in the postretirement years. In our study, concussion is defined as a blow (direct or transmitted) to the head resulting in symptoms such as headache, nausea, vomiting, dizziness/balance problems, fatigue, trouble sleeping, drowsiness, sensitivity to light or noise, blurred vision, difficulty remembering and difficulty concentrating (definition being clearly stated to the participants).^56^ In the case of concussion, additional information will be requested (eg, date, loss of consciousness, duration of recovery, hospitalisation). For these questions, participants are recommended to check their career timeline and to consult either their medical record or their physician.
- Brain structure and functioning: MRI sequences will be used to assess: (1) brain volume; (2) microstructural white matter integrity and (3) functional connectivity.^57 58^

Analysis of MRI data will be performed in FMRIB Software Library.^59^ Brain volumes (whole brain, white matter, grey matter and subcortical structures) will be assessed on conventional T1 scans using SIENAX and FIRST.^60 61^ White matter integrity will be assessed by means of fractional anisotropy and mean diffusivity extracted from diffusion tensor imaging, analysed with tract-based spatial statistics.^62^ Functional connectivity will be assessed by resting-state (participants instructed to close their eyes, relax their minds, not move and not fall asleep) functional MRI, using independent component analysis in MELODIC to extract and compare resting-state networks.^63^ Using 3 Tesla MRI scanners, MRI data acquisition will be performed according to standardised scanning protocols (table 1). Because this brain imaging technique is not likely to be available everywhere, only a subgroup of participants will be invited to this assessment.

**Table 1:** Scanning protocol for the assessment of brain volume, white matter integrity and functional connectivity

Domain	Scan type	Duration (min)
Preparation	Pilot and calibration	1
High-quality structural image	T1	3
White matter integrity	Diffusion tensor imaging	4
Functional connectivity	Resting-state functional MRI	6

- Neurocognitive functioning: Neurocognitive functioning will be assessed in several domains using the neuropsychological CNS Vital Signs (CNS-VS) online testing (OT) system (CNS Vital Signs, Morrisville, North Carolina, USA; https://www.cnsvs.com).^64^ Being available in many languages, the CNS-VS OT system has been previously used in various (professional) sports, including boxing, football and rugby.^65–68^ The psychometric properties of the CNS-VS tests were found to be very similar to the those of conventional neurocognitive testing, with moderate to good level of reliability (test–retest correlation coefficients: 0.65–0.88) and validity (concurrent validity correlation coefficients: up to 0.79).^69 70^ The CNS-VS tests were also found to discriminate between healthy subjects and patients with various psychological or neurological disorders.^71 72^ Seven neurocognitive domains will be assessed through seven CNS-VS subtests. The verbal memory test measures recognition memory for words, 15 words being presented, one by one, on the screen every two seconds. The participant has to identify those words nested among 15 new words (immediate recognition) while a delayed trial is conducted (delayed recognition). The visual memory test measures recognition memory for figures or shapes, 15 geometric figures being presented, one by one, on the screen. The participant has to identify those figures nested among 15 new figures (immediate recognition) while a delayed trial is conducted (delayed recognition). The finger tapping test (FTT) requires the participant to press the Space Bar with their right index finger as many times as they can in 10 s. After one practice run, the participant does three test trials and repeats FTT with the left hand. The symbol digit coding test consists of serial presentations of screens, each of which contains a bank of eight symbols above and eight empty boxes below. The participant types in the number that corresponds to the symbol that is highlighted. The Stroop test has three parts. In the first part, the words RED, YELLOW, BLUE and GREEN (printed in black) appear at random on the screen, and the participant presses the space bar as soon as he or she sees the word. In the second part, the words RED, YELLOW, BLUE and GREEN appear on the screen, printed in colour. The participant is asked to press the space bar when the colour of the word matches what the word says. In the third part, the words RED, YELLOW, BLUE and GREEN appear on the screen, printed in colour. The participant is asked to press the space bar when the colour of the word does not match what the word says. The shifting attention test measures the ability to shift from one instruction set to another quickly and accurately, using geometric objects with different shapes and colours. The continuous performance test measures the vigilance or sustained attention or attention over time. The participant is asked to respond to the target stimulus ‘B’ (randomly presented) but not to any other letter.

#### Cardiovascular health conditions

Cardiovascular health will be explored by collecting data on the following parameters: blood pressure (BP), lipid profiles and ECG abnormalities. These assessments related to cardiovascular health take around 15 min to be completed.

- BP: A mercury column sphygmomanometer, an appropriate-size cuff and a standard protocol is used to measure BP.^73^ Prior to the first of three measurements, participants are seated quietly in a chair with back support, with both feet flat on the floor for at least 5 min.^73^ BP is defined as the average of the second and third measurements, which are recorded more than 1 min apart.^73^ BP is interpreted according to the 2016 European Guidelines on cardiovascular disease prevention in clinical practice (<120/80 mm Hg as optimal; 120-129/80-84 mm Hg as normal; 130-139/85-89 mm Hg as high-normal; ≥140/90 mm Hg as hypertension).^73^
- Lipid profiles: Following a standard protocol, a venous blood sample (3–5 mL) is drawn in the morning after an overnight fast to obtain total cholesterol, high-density lipoprotein (HDL), low-density lipoprotein (LDL) and triglycerides (TGs). Dyslipidaemia is defined according to the 2016 European guidelines on cardiovascular disease prevention in clinical practice (eg, <2.6 mmol/L (<100 mg/dL) for LDL as high risk; >1.0 mmol/L (>40 mg/dL) for HDL and <1.7 mmol/L (<150 mg/dL) for TG as lower risk).^73^
- ECG abnormalities: A standard resting 12-lead ECG is recorded (not directly after physical activity) with the participant in the supine position during quiet respiration and recorded at 25 mm/s with a gain setting of 10 mm/mV.^74^ Abnormalities are defined according to the international criteria for electrocardiographic interpretation in athletes.^75^

### Independent and descriptive variables

According to the available aetiological evidence, the following risk indicators will be assessed at baseline and/or monitored over time (self-report, interview and/or physical examination): (family) history of cardiovascular disease, diabetes mellitus, medication and supplements use, body mass index (classified according to WHO), waist circumference, cigarette smoking status (two questions), level of physical activity (including maximal oxygen uptake in the pre-retirement years), employment status (postretirement) and (family) history of mental health disorder.^2 7 8 12 15 17 76 77^ Because salivary microRNAs (miRNAs) have recently emerged as promising biomarkers for neurocognitive diseases, saliva samples will be collected (baseline) in saliva collection pots which contain a proprietary miRNA stabilising solution (stored at the Amsterdam UMC, Amsterdam University Medical Centers (AMC)).^78^ The following descriptive variables will be assessed at baseline and/or monitored over time (self-report, interview and/or physical examination): age, height, body weight, percentage of body fat, professional football exposure (single questions about number and duration of matches and trainings), level of play, country of play, educational level, health-related quality of life and cardiac events.^79^

### Data collection, storage, interpretation and sharing

Baseline and follow-up data in our study will be collected through the following three sources:

- Football-related medical assessment (FMA) conducted yearly or biennially in the context of the precompetition medical assessment in professional football (according to standard protocols) by a medical professional (eg, physician, cardiologist, radiologist), including history taking, physical examination, cardiac screening and/or imaging.^80^
- Study-related medical assessment (SMA) conducted specifically in the context of the study (according to standard protocols) by a medical professional (eg, physician, cardiologist, radiologist), including history taking, physical examination, cardiac screening and/or imaging.
- OT completed specifically in the context of the study, being set up within a secured online system (CastorEDC, CIWIT B.V, Amsterdam, the Netherlands).

**Figure 1:**
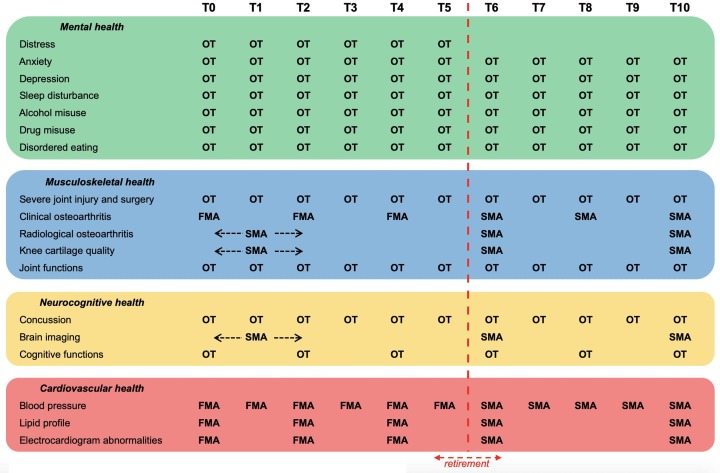
Overview of the data collection about the mental, musculoskeletal, neurocognitive and cardiovascular health of professional footballers. FMA, footbal-related medical assessment; OT, online testing; SMA, study-related medical assessment.

In the preretirement years, data for all variables except for brain imaging, OA and cartilage quality (feasibility reasons) will be assessed every year or every second year through FMA (start of football season), SMA (start of football season) and/or OT (start of calendar year). In the postretirement years, data for all variables except for lipid profile, brain imaging, OA and cartilage quality (feasibility reasons) will be assessed every year or every second year through SMA (mid-calendar year) and/or OT (start of calendar year). The expected timeline and source of data collection for all health conditions (dependent variables) is presented in figure 1. Linked to participants through a unique identifier, all data collected for this study will be pseudonymised and stored on the secure server of the location AMC. Only the two co-principal investigators (PIs) will have access to subject-identifiable information while they will regulate access to the data and be responsible for maintaining confidentiality and data protection. The interpretation of the collected data will be the responsibility of the authors with the relevant expertise. Collected data will remain the property of the two co-PIs, and will be shared with researchers who provide a methodologically sound proposal.

### Study sample and recruitment

All members of FIFPRO and affiliated national unions in England, Finland, France, the Netherlands, Norway, Sweden and Switzerland fulfilling the inclusion criteria (n=750) will be invited for the study. Expecting a response rate of 40% and a loss to follow-up at 10% per year, we will enrol at least 200 participants at baseline. With regard to the outcome measures under study and with 95% CI, we will achieve more than 80% statistical power to detect within a precision of 10% that 2 out of 10 participants will suffer from a health condition (eg, symptoms of anxiety/depression, knee OA).^81 82^ Participants will be recruited by FIFPRO in one or two stages, the first stage being through the 2019–2020 season. Eligible participants will receive thorough information about the study’s aim and procedures (in English and native languages). If interested, participants will give their informed consent and be enrolled in the study. If the intended sample size is not achieved after the 2019–2020 season, a second stage will be planned through the 2020–2021 season. Before the start of the study (June 2019), the intended recruitment of participants (as well as the whole methodology) was discussed in detail with the national players’ unions involved during a meeting in Hoofddorp, the Netherlands (FIFPRO’s headquarters).

### Matched controls from the non-elite sport population

Participants (professional footballers) enrolled in the study will be asked to find in their social network from a non-elite sport population one control matched for gender, age, height and body weight. Eligible matched controls will receive thorough information about the study and give their informed consent if interested. Matched controls will then be asked to complete all OT assessments (see figure 1), collected data being pseudonymised and stored on the secure server of the AMC. For all FMA and SMA (figure 1), data from the participants (professional footballers) will be compared with data from existing registers or studies (matched controls from non-elite sport population).

### Statistical analyses

All data analyses will be conducted by using the statistical software IBM SPSS Statistics. Descriptive data analyses (eg, mean, SD, median, frequency) will be performed at baseline and during follow-up for all variables involved in our study. For our descriptive objective, prevalence and incidence will be calculated for all health conditions (dichotomous; dependent variables), using the adjusted Wald method (sample size of 150 persons or less) or the Wald method (sample size of more than 150 persons) for 95% CI.^82^ Prevalence (expressed as a percentage) will be calculated as the proportion of the number of participants with a given health condition relative to the total number of participants.^82^ Incidence (expressed as a percentage) will be calculated as the proportion of the number of participants with a newly given health condition during the follow-up relative to the total number of participants free from this health condition at baseline.^82^ Comparison of the onset and course of the health conditions under study between professional footballers and the matched controls from the non-elite sport population will be explored with X^2^ (dichotomous data) and independent t-test (continuous data).^82^ For our analytical objective, descriptive data analyses (eg, mean, SD, median, frequency) of all data (dependent and independent variables) over time will be conducted (within participants and between subgroups of participants) and differences between subgroups of participants will be assessed with X^2^ (dichotomous data) and independent t-test (continuous data).^82^ Mixed-effects (logistic) regression (maximum likelihood estimation) will be used to explore potential associations between risk indicators (independent variables) and health conditions (dependent variables) over time (table 2).^83^

**Table 2:** Potential associations between risk indicators and the health of professional footballers

Domain	Risk indicator
Mental health	Family history Severe injury Surgery Concussion Employment status
Musculoskeletal health	Severe injury Surgery Body mass index
Neurocognitive health	Family history Concussion
Cardiovascular health	Family history Diabetes mellitus Medication and supplements use Body mass index Waist circumference Cigarette smoking status Level of physical activity

### Patient and public involvement

Participants were not directly involved in defining the objectives and design of the study. However, participants’ representatives were consulted and provided input to the recruitment’s strategy.

## Ethics, dissemination and discussion

Prior to enrolment in the study, eligible participants will sign an informed consent. Participants will participate voluntarily, being enable to withdraw from the study at any time without prejudice. In order to facilitate adherence of the participants to the study and thus longitudinal data collection, every second year of the follow-up, players will receive a general report about their mental, musculoskeletal, neurocognitive and cardiovascular health (including potential advice regarding standard care). In the case of results with potential adverse health consequences, one of the PIs will discuss the implications of these results with the participant concerned, as well as his potential need to inform his club physician and/or General Practitioner. Club physicians and/or general practitioners will be notified only after the consent of the participants concerned. All participants will be welcome to discuss any results with one of the PIs at any time. The results of the study will be submitted to peer-reviewed scientific journals for publication, as well as being presented at scientific conferences and released in the media (postpublication).

To the authors’ knowledge, this is the first prospective cohort study to gather epidemiological insight (descriptive and analytic) about the mental, musculoskeletal, neurocognitive and cardiovascular health of professional footballers during their preretirement and postretirement years. Currently, there is some evidence available in isolation about a specific health domain in either active or retired players, but a longitudinal approach across health domains (mental, musculoskeletal, neurocognitive, cardiovascular) is lacking. Monitoring the health of professional footballers before, through and after their transition out of sport will allow us to: (1) gather evidence about musculoskeletal, neurocognitive, cardiovascular and mental health trends during their preretirement and postretirement years; (2) identify fundamental predisposing (risk) factors for potential short-term and long-term adverse health consequences of a professional football career; and (3) get a better understanding about when and how adverse adaptations might occur and when support measures should be offered. Ultimately, this will enable the development of preventive and therapeutic support measures in order to empower the health of professional footballers, leading to their healthier transition out of sport and to a higher quality of life.

The four major challenges of our prospective cohort study are related to: (1) enrolment of participants (professional footballers); (2) recruitment of matched controls from a non-elite sport population and accessibility to data from existing registers or studies; (3) potential unexpected costs and (4) cohort retention during the follow-up period. A total of at least 200 professional footballers, aged around 27 years, will be enrolled in the study. Therefore, FIFPRO’s affiliated national unions from seven countries were approached due to their positive past experiences regarding health-related studies and due to their high rate of membership among professional footballers. This should facilitate significantly the enrolment of at least 200 participants, especially with regard to the 750 players aged 27 years being available in these countries. The recruitment of matched controls will rely on the social network of the enrolled participants, a strategy used successfully in a previous study of the two co-PIs. Although this prospective cohort study has been designed and prepared logistically and financially for nearly 12 months, we cannot exclude the possibility that some unexpected costs will occur during the follow-up period of 10 years. In that case, the authors will strive to secure additional funding for these costs. Since cohort retention is a challenge for any prospective cohort study,^84^ several strategies will be used in our prospective cohort study in order to secure participants’ adherence and maintain planned data collection. First, participants will be kept informed and updated by FIFPRO and affiliated national unions in English and respective native language. Second, information will be sent to participants by email and social media. Third, the main contact persons for the participants (co-PIs and FIFPRO’s affiliated national unions) will remain consistent over the study period. Lastly, when they actually retire from football, all participants will be offered the After Career Consultation recently developed and positively assessed in a pilot implementation.^85^
